# Minimal regulatory spaces in yeast genomes

**DOI:** 10.1186/1471-2164-12-320

**Published:** 2011-06-16

**Authors:** Wei-Hua Chen, Wu Wei, Martin J Lercher

**Affiliations:** 1 European molecular biology laboratory (EMBL), Meyerhofstrasse 1, 69117 Heidelberg, Germany; 2 Institute for Computer Science, Heinrich-Heine-University Düsseldorf, Universitätsstr. 1, 40225 Düsseldorf, Germany

## Abstract

**Background:**

The regulatory information encoded in the DNA of promoter regions usually enforces a minimal, non-zero distance between the coding regions of neighboring genes. However, the size of this minimal regulatory space is not generally known. In particular, it is unclear if minimal promoter size differs between species and between uni- and bi-directionally acting regulatory regions.

**Results:**

Analyzing the genomes of 11 yeasts, we show that the lower size limit on promoter-containing regions is species-specific within a relatively narrow range (80-255 bp). This size limit applies equally to regions that initiate transcription on one or both strands, indicating that bi-directional promoters and uni-directional promoters are constrained similarly. We further find that young, species-specific regions are on average much longer than older regions, suggesting either a bias towards deletions or selection for genome compactness in yeasts. While the length evolution of promoter-less intergenic regions is well described by a simplistic, purely neutral model, regions containing promoters typically show an excess of unusually long regions. Regions flanked by divergently transcribed genes have a bi-modal length distribution, with short lengths found preferentially among older regions. These old, short regions likely harbor evolutionarily conserved bi-directionally active promoters. Surprisingly, some of the evolutionarily youngest regions in two of the eleven species (*S. cerevisiae *and *K. waltii*) are shorter than the lower limit observed in older regions.

**Conclusions:**

The minimal chromosomal space required for transcriptional regulation appears to be relatively similar across yeast species, and is the same for uni-directional and bi-directional promoters. New intergenic regions created by genome rearrangements tend to evolve towards the more narrow size distribution found among older regions.

## Background

Expression of a gene requires a functional promoter region 5' of the coding sequence. Promoter regions encompass the binding sites for DNA polymerase, usually accompanied by *cis-*regulatory sequences. It is likely that this regulatory information - including spacers and the 5' untranslated region (UTR) - needs a minimum length of DNA sequence. In most cases, promoter regions will not overlap with the coding or UTR regions of neighbouring genes, both because of functional constraints and because transcription of the neighbour gene would interfere with transcription initiation at the promoter [1,2]. A lower size limit for promoter regions can thus be obtained from the length distribution of promoter containing regions located between the coding sequences of gene pairs (inter-CDS regions).

**Figure 1:**
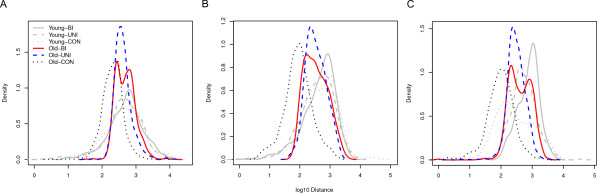
**Size distribution of old and young inter-CDS regions in different yeast species**. A) *Saccharomyces cerevisiae*, B) *Kluyveromyces waltii*, C) *Zygosaccharomyces rouxii*. Figure legends are: Young - interCDS regions that are not conserved in any other ten yeast species; Old - interCDS regions that are conserved in any other ten yeast species; BI - inter-CDS regions flanked by bidirectional/divergent gene neighbours; UNI - inter-CDS regions flanked by unidirectional/co-oriented gene neighbours; CON - inter-CDS regions flanked by convergent gene neighbours. See Additional file 1 for the remaining eight species.

We can classify inter-CDS regions according to the relative orientation of the two genes flanking the region. First, the two genes can be located on the same strand, and will thus be transcribed uni-directionally (→→ or ←←, Figure 1). This type of inter-CDS region will contain the promoter for one gene, which flanks the region with its 5' end. Second, the two genes can be transcribed divergently (←→), with the promoter region(s) of both genes located in the inter-CDS region. The two flanking genes can have two fully independent promoter regions, or one shared promoter region may act bi-directionally [3-5]. Finally, the two flanking genes may be transcribed convergently (→←), and in this case the inter-CDS region does not contain a promoter. When examining our hypothesis of a minimal regulatory/promoter space, the size distributions of these promoter-less 'convergent' regions can be used as a Null expectation. If non-functional yeast DNA tends to be removed either by selection or by a mutational bias [6], then convergent inter-CDS regions should on average be shorter than either 'uni-directional' or 'divergent' regions, and they should not have a 'hard' lower limit.

In genomes that contain many repetitive and non-functional sequences, such as the human genome, few inter-CDS regions come close to the lower limit indicative of a minimal promoter space. For our question it is hence desirable to study compact genomes. Unicellular yeasts are ideal for this purpose, as a large number of yeast genomes has been sequenced, and as these species typically have small genomes with short inter-CDS regions. Below, we therefore concentrate on *Saccharomyces cerevisiae *and its relatives.

Genomic rearrangements may often create inter-CDS regions that are substantially shorter or longer than the inter-CDS regions that were broken up. If a resulting promoter region is too short, this will result in functional impairment (e.g., because individual sites involved in polymerase binding are missing or are too close to each other). It is conceivable that sometimes this impairment is outweighed by accompanying expression changes that are advantageous [7], and such an imperfect promoter region may still spread through the population. In such cases, we expect that the promoter region will be extended through insertions over evolutionary time to regain optimal functionality. Conversely, newly created regions may also be unusually long; while this may not affect promoter function, we expect a length reduction over time, either due to a mutational bias towards deletions [6] or due to selection for genome compactness. Overall, we expect that evolutionarily young inter-CDS regions may have a different length distribution compared with older regions [1]. We therefore focus on old inter-CDS regions throughout most of this study, which are more likely to represent the equilibrium size distribution.

By far the strongest predictor of gene neighbourhood conservation is intergenic or inter-CDS distance [8]. This is consistent with a purely mechanistic (non-selective) model of rearrangements, and indicates that most rearrangements between genes are neutral. Under this model, long regions are most likely to disappear during evolution, and the size distribution of inter-CDS regions should evolve towards an equilibrium state. Below, we find that a very simple neutral model is indeed capable of explaining the variation of length distributions with evolutionary age of the convergent inter-CDS regions, but fails to fully capture the dynamics of promoter containing regions in most species.

In this paper, we thus analyse the length distributions of inter-CDS regions across yeast species to examine the following questions: (i) are promoter-containing regions longer than promoterless regions, indicating a minimal promoter length?; (ii) does minimal promoter size differ between uni-directional and divergent regions?; (iii) does minimal promoter size differ between species?; and (iv) can the size distribution of inter-CDS regions be explained by a simplistic neutral model of rearrangements?

## Results

We identified inter-CDS regions flanked by protein coding genes in 11 fully sequenced and well-annotated yeast species [9,10]. Regions flanked by tandemly duplicated genes may be under special forms of selection [11] and may hence evolve differently; these regions were removed from further analysis.

Inter-CDS regions that were recently formed by genomic rearrangements may not have had time to evolve towards their 'optimal' length [1]. For each examined yeast genome, we therefore focused first on ancestral ('old') regions, i.e., those that have an orthologous inter-CDS region in another yeast species (see Materials and methods); all other inter-CDS regions are labelled 'young'.

### A narrow, species-specific length distribution in inter-CDS regions lacking promoters

It is generally assumed that most *cis*-regulatory sequences are organized into promoter regions located directly 5' of their regulated genes. Accordingly, few *cis-*regulatory sequences should be found in inter-CDS regions flanked by the 3' ends of convergently transcribed genes. We thus expect that such 'convergent' inter-CDS regions have no hard limits on their size distribution. Furthermore, if DNA insertions and deletions were selectively neutral and unbiased in these regions, then length changes per unit time via indels should be proportional to the region length (as each nucleotide would have the same chance of being an insertion or deletion point). On log-scale, such random length changes over time would be additive, and we hence expect a log-normal distribution of lengths under these conditions. In agreement with this expectation, Figure 1 and Additional file 1 show approximately log-normal length distributions for old convergent regions in all 11 examined yeast species.

Mean inter-CDS length of convergent pairs is fairly similar across species, ranging from 153 bp in *Kluyveromyces waltii *to 316 bp in *Saccharomyces bayanus *(Table 1). Lengths within one species typically vary by a factor of two (standard deviation 0.30-0.45 on the log_10_-scale). This relatively narrow range indicates that each yeast has its species-specific equilibrium length of convergent regions. A 'soft' lower limit >0 bp is likely due to the 3' UTRs, and may further be enforced by the avoidance of transcriptional interference between the flanking genes (see [2] and references therein). An equally soft upper limit may result from a bias towards deletions (rather than insertions) in non-functional DNA [6], but may also stem from a higher probability of rearrangements in longer regions [8] (see below).

**Table 1:** Summary statistics for the inter-CDS length distributions across 11 yeast species.

Species	cat		old			young	
		divergent	unidirectional	convergent	divergent	unidirectional	convergent
sce	Mean *	489	412	205	486	453	415
	location of lower limit **	172	159	49	27	12	19

ago	mean	322	291	106	531	416	227
	location of lower limit	78	63	9	59	58	10

cgl	mean	616	606	248	918	752	354
	location of lower limit	206	242	64	178	189	99

kla	mean	613	513	172	904	686	327
	location of lower limit	199	193	19	186	158	29

kpo	mean	853	725	213	1496	896	339
	location of lower limit	255	265	40	335	166	83

kth	mean	427	360	103	968	788	465
	location of lower limit	84	97	10	154	91	48

kwa	mean	345	340	88	433	370	464
	location of lower limit	88	74	8	17	18	16

sba	mean	518	438	217	1194	1071	909
	location of lower limit	173	165	52	168	194	153

sca	mean	380	360	158	643	467	231
	location of lower limit	122	130	43	129	118	39

skl	mean	412	445	155	920	745	511
	location of lower limit	95	111	18	111	65	36

zro	mean	408	312	96	750	538	273
	location of lower limit	100	85	9	138	95	30

* mean lengths were calculated as 10^(average(log10( inter-CDS lengths)));

** location of lower limit is defined by 2% quantile of the inter-CDS length distribution in each category.

### A lower limit on the length of promoter-containing regions

If successful transcription initiation and regulation requires a minimal set of *cis-*regulatory nucleotides, we expect that regions neighbouring at least one 5' CDS end require a minimal length. Consistent with this expectation, Figure 1 and Additional file 1 show that in all examined species, there are practically no old uni-directional or divergent inter-CDS regions shorter than approximately 130 bp (see Table 1 for summary statistics of the length distributions and Additional file 2 for statistics of underrepresentation of old uni-directional and divergent pairs at short distances). This lower size limit appears to be species-specific within a relatively narrow range, varying between approximately 80 bp and 260 bp.

Interestingly, across all species, the lower size limit is very similar between uni-directional and divergent inter-CDS regions (Figure 1, Additional file 1 and Table 1). This is consistent with the existence of bi-directional promoters in yeast [5], which initiate transcription in both directions. Our results then suggest that minimal bi-directional promoters require the same space as do minimal uni-directional promoters.

In each species, promoter-containing regions are on average at least twice as large as convergent inter-CDS regions; in some cases, the lower size limit of promoter-containing regions coincides with the peak of the convergent length distribution. This difference supports the requirement of promoter-binding and *cis-*regulatory sites as the cause for the minimal length of promoter-containing regions, and hence justifies our approach *a posteriori*.

The length distribution of old uni-directional inter-CDS regions can be approximated reasonably well by a log-normal distribution, with two small deviations (Figure 1 and Additional file 1). The left slope of the logarithmic length distribution, which corresponds to the minimal length, is usually steeper than the right slope; the latter probably does not reflect a hard limit. Furthermore, the right tail is elevated, indicating an excess of longer regions. It is possible that these long regions harbour unusually large numbers of *cis-*regulatory nucleotides, or that they contain other un-annotated functional sequences.

### Similar length distributions for all but the youngest inter-CDS regions

The age of inter-CDS regions can be approximated by the divergence time of the last common ancestor of the query genome and the most distal genome with an orthologous inter-CDS region. We reconstructed the phylogenetic relationships of the 11 yeast genomes using *Schizosaccharomyces pombe *as an outgroup (Additional file 3), and then classified the *S. cerevisiae *inter-CDS regions into three age groups (see Materials and methods).

**Figure 2:**
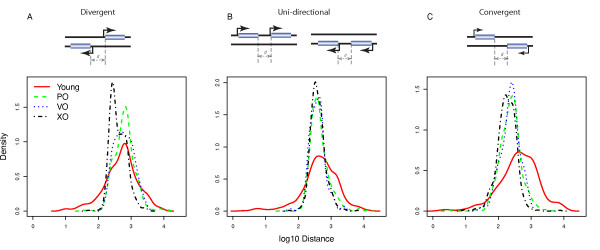
**Minimal length of inter-CDS regions doesn't increase with the age of the regions**. Depending on the phylogenetic distance to orthologous neighbouring gene pairs, we classified inter-CDS regions in *S. cerevisiae *into four age groups: 'young', 'plain old' (PO), 'very old' (VO), and 'extra old' (XO) (see Methods). (A) regions flanked by divergently transcribed genes; (B) regions flanked by uni-directionally transcribed genes; (C) regions flanked by convergently transcribed genes. The length distributions of all three 'old' age groups are very similar, except for the 'divergent' regions, where the relative weight shifts from the higher to the lower peak with increasing age.

As shown in Figure 2, the minimal length of promoter-containing regions is very similar across all but the youngest age groups, suggesting that this minimum length is maintained by selection. The youngest age group shows a much broader length distribution, suggesting that these regions did not have enough time to evolve towards the equilibrium distribution seen for older regions. Consistent with Figure 1, the minimal length appears to be very similar between uni-directional and divergent regions in each age class.

### Minimal inter-CDS length is correlated with genome size and total intergenic space

In the presence of either a bias towards deletions or selection for genome compactness, we expect a strong correlation between total intergenic space and minimal promoter size. Furthermore, as genome size is the sum of gene-coding and intergenic space, we also expect minimal promoter size to be correlated with genome size. Indeed, minimal promoter length across the 11 yeast species is well predicted by total intergenic space (Pearson's correlation coefficient *R *= 0.87, *p *= 0.00058) and by genome size (*R *= 0.81, *p *= 0.0025). In contrast, we find no significant correlation between minimal promoter length and the number (*R *= -0.21, *p *= 0.54) or total length (*R *= 0.22, *p *= 0.52) of protein-coding genes.

### Two types of divergently transcribed regions with different conservation

While the logarithmic length distributions of both the uni-directional and the convergent regions can be approximated reasonably well by a single Gaussian, this is not the case for the divergent regions. The latter appears bi-modal, and is fitted significantly better with two independent Gaussians (ANOVA: *P *< 10^-100 ^for the logarithmic length distribution of old divergent regions in each species, comparing fits of 1 and 2 Gaussians; see Additional file 4). The left peak roughly coincides with the single peak of the uni-directional regions, suggesting that it may contain a single promoter region that acts bi-directionally. Conversely, it appears likely that the longer divergent regions contain two (at least partially) independent promoter regions.

Interestingly, while the minimal length does not vary between age groups, the relative weight of the two peaks for divergent regions shifts from longer regions to short regions with increasing age (Figure 2A). If the two peaks indeed correspond to bi-directional and independent promoters, this suggests that gene pairs linked by a bi-directional promoter are strongly conserved in evolution, as expected. This observation is consistent with previous studies, which found that most highly conserved inter-CDS regions in fungi tend to be bi-directional [12].

### Young promoter-containing regions are also rarely short in most species - *S. cerevisiae *and *K. waltii *being notable exceptions

So far, we have restricted our analyses to old inter-CDS regions, i.e., those for which an orthologous region can be found in another yeast species. We now switch our attention to young (species-specific) inter-CDS regions. The length distributions of promoter-containing young regions also respect the minimal length derived from old inter-CDS regions in all species - with the exception of *S. cerevisiae *and *K. waltii *(Figure 1 and Additional file 1). Both of the latter species show an excess of very short regions (<100 bp).

Due to the inclusion of close relatives of *S. cerevisiae *and *K. waltii *(*Saccharomyces bayanus *and *Kluyveromyces thermotolerans*, respectively - see Additional file 3) in our study, 'young' genes in these two species are indeed younger than 'young' genes in the other species examined, except for the sibling species themselves. Thus, if selection is responsible for the removal or extension of short upstream regions, it may simply be that selection has not had enough time to act in these very young sets of inter-CDS regions (although in this case we might expect similar patterns in the sibling species). Furthermore, a recent population expansion in *S. cerevisiae *during its co-evolution with human agriculture [13] may have reduced selection against slightly deleterious short upstream regions in this species.

### Young regions are more variable in size and tend to be longer

As evident from Figure 1, young inter-CDS regions are more variable in size and tend to be longer [1], regardless of gene orientation (see also Additional file 1 and Table 1). Thus, it appears that genomic rearrangements often create inter-CDS regions that are longer than the genomic mean. To reach the observed distribution of older regions over evolutionary time, these new, long regions must disappear again. This can either happen through the deletion of non-functional nucleotides, or through renewed rearrangement of the long regions.

It is noteworthy that the length difference between young and old inter-CDS regions tends to be larger for divergent compared to uni-directional regions. This is likely a consequence of the fact that most newly created divergent regions contain two (largely) independent promoter regions; conversely, old regions often contain one bi-directional promoter (Figure 2), and hence require less space on average.

### A simple neutral model of distance-dependent rearrangements

In a careful study, Poyatos and Hurst [8] found that by far the strongest predictor of gene neighbourhood conservation in yeasts was inter-CDS length. This suggests a predominantly neutral model of gene rearrangements, where non-homologous recombination is approximately equally likely at each (non-functional) nucleotide. Such a simple model necessarily ignores the importance of genomic neighbourhoods; *e.g*., neighbouring genes may be co-regulated through changes in chromatin state [14,15], or may be co-localised in regions of high or low recombination rate [16]. However, it may be that these neighbourhood effects are the exception rather than the rule [8], and it hence appears fruitful to examine if the simple neutral model is able to explain the observed differences between the length distributions of young and old inter-CDS regions.

Thus, we employed a simulation in which the probability that an inter-CDS region is broken up depends only on its length. We start from the total observed length distribution (combining old and young regions) of a given species, using this as an approximation to the total length distribution in the common ancestor of the species and its closest relative in our dataset. We then test if random rearrangements can reduce this total 'ancestral' distribution to the distribution seen for *old *regions now (*i.e.*, those regions that survived from the common ancestor). In each time step, we randomly chose an inter-CDS region and removed it from the distribution with a probability proportional to its length; this simulates rearrangements, where ancestral regions are destroyed to create new regions. This random removal was repeated until the number of rearrangements was identical to the lower bound on the number of rearrangements in the real data, i.e., until we removed *N*_young _regions. It has to be pointed out that our model is very simplistic, not only in its assumption that rearrangements are strictly proportional to the length of the inter-CDS region. Importantly, length changes also occur through insertions and deletions; these are ignored here.

**Figure 3:**
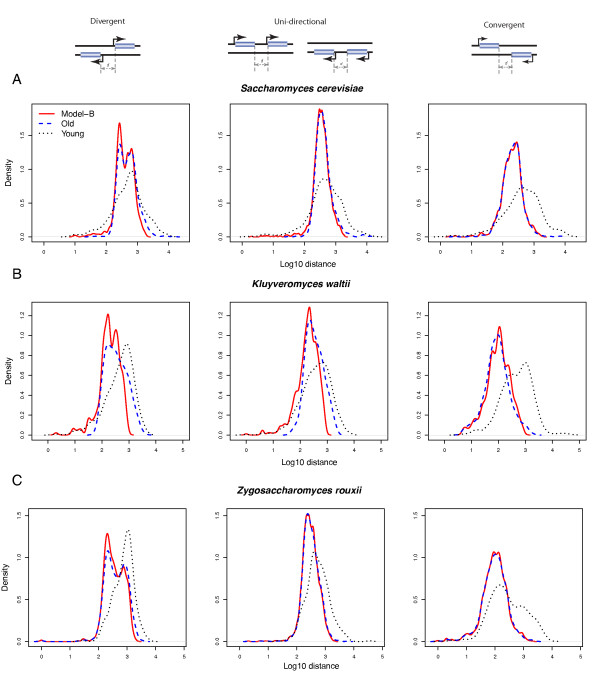
**Modelling-B of size distribution of inter-CDS regions in different yeast species**. Left column: between divergently transcribed genes; middle column: between co-oriented genes located on the same strand; right column: between convergently transcribed genes. Length distributions are shown separately for young gene pairs (those without neighbouring orthologs in any other yeast species) and for older gene pairs. Red lines are predictions for the 'old' distributions from a simulation of neutral rearrangements of the total distribution (see Materials and methods for details, and see Additional file 6 for an alternative neutral model). See Additional file 5 for the remaining eight species.

If the model approximately reflects the underlying biology, then the simulated length distribution should closely match the length distribution of old inter-CDS regions. As evident from Figure 3 and Additional file 5, this is indeed the case for convergent regions. In contrast, the fit is less good for the two types of promoter containing regions in most species. In almost all species, the observed old length distributions show an excess of long inter-CDS regions that is not captured in the simulations (Figure 3 and Additional file 5; see also Additional file 6 where the model doesn't require an 'unbreakable' promoter region). Thus, some long regions appear to be maintained by stabilising selection. In addition, *S. cerevisiae *and *K. waltii *show a dearth of very short regions in the real data compared to the simulations. This suggests that either the real ancestor did not have a similar number of very short young regions as the extant species, or that these regions were preferentially removed or extended by natural selection.

### The distribution of repeat regions does not bias our results

Repeat-sequences (*e.g.*, TY elements) in inter-CDS regions may serve as hot-spots for non-homologous recombination. Their presence may therefore modify gene neighbourhood in a non-random, non-selective manner. However, all results presented above remain essentially unchanged after excluding repeat-containing inter-CDS regions identified using RepeatMasker [17] (data not shown). Thus, genomic repeats appear not to be driving the evolution of intergenic length distributions.

## Discussion

Promoter-containing inter-CDS regions harbour *cis-*regulatory elements that are essential for transcriptional control and initiation. The required number of *cis-*regulatory nucleotides (including spacers) may vary according to the complexity of expression patterns among genes. However, this will depend on how expression signals are integrated along the hierarchy of transcriptional regulators. For example, a transcription factor may be induced according to complicated combinatorial cues by a variety of environmental signals. This single transcription factor may then simply transfer these integrated signals to all its regulatory targets - all the *cis-*regulatory complexity is then accounted for by the promoter region of the transcription factor, allowing for complex expression patterns with simple *cis-*regulatory sites for the majority of genes.

We therefore expect that many genes require little more than a minimal promoter configuration (although some genes still require complicated regulation, consistent with the slight excess of very long regions compared to our simple simulations). However, it appears highly likely that successful (and orderly) initiation of transcription still requires a certain minimum of *cis-*regulatory sites. That this is indeed the case is evidenced by the length distributions of inter-CDS regions in Figure 1 and Additional file 1, which appear truncated at low distances for uni-directional and divergently transcribed regions, *i.e.*, those regions containing promoters.

Many eukaryotic promoters initiate transcription on both strands, even if transcription often stalls in one direction [3,4]. Consistent with this observation, we find that the length distribution of divergent regions is bi-modal, with a shorter peak that roughly co-incides with the single peak of uni-directional regions. These peaks likely represent optimized regulatory spaces for shared promoters in divergent pairs and single promoters in uni-directional pairs, respectively. The coincidence of the two peaks results in very similar minimal inter-CDS lengths in the two types of regions.

**Figure 4:**
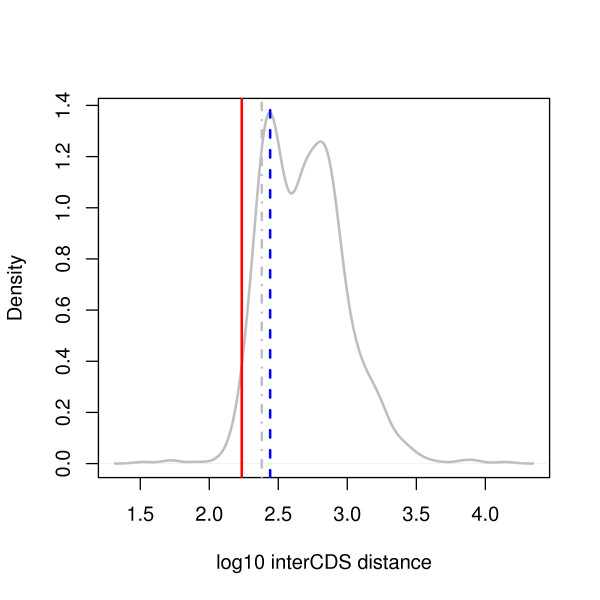
**Minimal length is shorter than the optimized regulatory space**. Shown here is the length distribution of divergent regions in *S. cerevisiae *that are conserved in other yeast species. The minimal length of 172 bp is defined by the lower limit of the length distribution (Table 1) and plotted in red; the coincidence of the first peak with a canonical promoter space of 140 plus two times of median length of 5'UTRs (blue vertical line) defines putative optimized regulatory space and is longer than the minimal distance. This optimized space is slightly shorter if it's calculated using another source of 5'UTR median length (grey vertical line) [20] but still longer than 172 bp.

In *S. cerevisiae*, a canonical promoter of ~140 bp upstream to transcription start sites (TSS) was previously described [18]. This distance plus two times the median 5'UTR length (68 bp [19]) coincides perfectly with the first peak of the old divergent regions (those that are conserved in other yeast species; Figure 4), supporting our interpretation that the peak indeed represents the optimized regulatory space. We obtained a similar size of optimized regulatory space using 5'UTR median length published in [20] (Figure 4, grey vertical line). Not surprisingly, this optimized distance (140 + 68*2 = 276 bp) is somewhat longer than the minimal space of 172 bp we identified in *S. cerevisiae*, which represents the lower limit of a functional promoter space.

What is puzzling is that in two species, *S. cerevisiae *and *K. waltii*, some evolutionarily young gene pairs get away with much shorter promoter regions (Figure 1). These are not pseudogenes, as there is unequivocal evidence for their expression (data not shown). It is conceivable that despite some functional impairment, these recently created short promoter regions might still provide a selective advantage (*e.g.*, by elevating co-expression between neighbours, or by disturbing the expression of one gene by its neighbour [7,21]); in this case, we would expect the promoter regions to grow over time to allow better transcription initiation and/or regulation. Conversely, it is possible that the short promoter regions present in the published genome sequences are in fact slightly deleterious; they may have survived due to population expansions (as during the co-evolution of *S. cerevisiae *with human agriculture), or they may even be polymorphic in the respective populations.

## Conclusion

In this study we show that the minimal chromosomal space is required for transcriptional regulation in yeast species; while the minimal length varies between yeast species, this variation is relatively small. The position of the left edge of the distribution varies between 78 bp and 255 bp (Figure 1, Additional file 1, and Table 1). Subtracting the mean length of promoter-less inter-CDS regions in each species (Table 1) results in an estimate of minimal promoter length of 54-225 bp. Note that the average size of promoterless convergent regions has a similar coefficient of variation across species (standard deviation/mean = 0.35) as uni-directional (0.32) and bi-directional regions (0.30); this similarity suggests that the observed differences across species may reflect different patterns of genome evolution rather than differences in promoter organisation. Thus, yeast promoters appear to require a minimal length of 115 ± 50 bp, regardless of whether they initiate transcription on one or on both strands.

## Methods

### Sequences and inter-CDS regions

We downloaded protein sequences and genome annotations for eleven yeast species (*Kluyveromyces lactis *, *Ashbya gossypii*, *Saccharomyces kluyveri*, *Kluyveromyces waltii*, *Kluyveromyces thermotolerans*, *Zygosaccharomyces rouxii*, *Kluyveromyces polysporus*, *Candida glabrata*, *Saccharomyces castellii*, *Saccharomyces cerevisiae*, *Saccharomyces bayanus*) from the Yeast Gene Order Browser [9,10]. We defined neighbouring gene pairs as protein-coding genes that are direct chromosomal neighbours, with no intervening genes and no overlap of the transcripts. We grouped gene pairs (and the intervening inter-CDS regions) into one of three types according to the relative orientations of the genes: convergent (→←), uni-directional (→→ or ←←), and divergent (←→) [2]. We calculated inter-CDS distances using open-reading frame (ORF) boundaries. We identified putative tandem duplication genes based on BLAST searches and removed those pairs with bitscore ≥50.

### Age of inter-CDS regions

We used INPARANOID [22] to identify pairwise orthologous relationships for protein sequences between any two yeast genomes, and retained orthologous groups with 100% bootstrap support for subsequent analysis.

For each of the 11 species, we then searched ancestral inter-CDS regions in the other 10 species. We considered a region conserved only if both flanking genes had orthologs in the other genome, and if those orthologs were also direct neighbours in the same orientation; the corresponding inter-CDS regions were then considered orthologs.

To reconstruct the phylogenic relationships among downloaded yeast species, we selected ~40 universal single-copy marker genes previously suggested by Ref. [23], and aligned each ortholog set using MUSCLE [24]. We concatenated the resulting multiple-sequence alignments, elimated poorly aligned and divergent regions using Gblocks [25], and then used the maximum-likelihood approach implemented in PHYML [26] to reconstruct the phylogenetic tree. We used the parameters described in Ref. [23] for all programs. To root the tree, we used *Schizosaccharomyces pombe *[27] as an outgroup; we downloaded its protein sequences from Sanger (http://www.sanger.ac.uk/Projects/S_pombe/) in April 2010. The resulting tree is shown in Additional file 3.

We then classified the inter-CDS regions in *S. cerevisiae *into four age groups, according to the most distal genomes in which conserved neighbourhood was found. 'young' regions are those without conservation in any other species; 'plain old' (PO) are regions with orthologs in *S. bayanus *or *S. castellii*; 'very old' (VO) are regions with orthologs in any of *C. glabrata*, *K. polysporus *and *Z. rouxii*; and 'extra old' (XO) are those regions with orthologs in any of *K. thermotolerans*, *K. waltii*, *S. kluyveri*, *A. gossypii *and *K. lactis *(see Additional file 3 for more detailed information on their phylogenetic relationships).

### Modelling long-term genome rearrangements

Intergene distance is by far the strongest predictor of gene neighbourhood conservation in *S. cerevisiae *[8]. This is consistent with a simple neutral model in which rearrangements are equally likely at each inter-CDS nucleotide. We performed simulations to test if this model is sufficient to explain the difference in the length distributions of young and ancestral inter-CDS regions even when ignoring other factors contributing to length evolution.

We used two versions of the model: in Model A (results shown in Additional file 6), the probability that a neighbouring gene pair is broken up depends only on the total distance between the coding sequences. In Model B (shown in the main text), we assume that rearrangements within the promoter region are strongly deleterious, and that rearrangements therefore only occur at nucleotides outside the promoter region. To approximate this effect, we assume that rearrangement probability is proportional to the length of the inter-CDS region minus the minimal promoter length in Table 1 ('corrected' length).

In a given genome, we have *N*_*total *_= *N*_young _+ *N*_*old *_inter-CDS regions. On the terminal branch of the phylogenetic tree, *N*_young _new pairs were formed by breaking up the same number of ancestral (old) pairs. We assume that the inter-CDS length distribution is in equilibrium, i.e., the length distribution at the ancestral node was identical to the total length distribution observed now. We then set the probability that each inter-CDS nucleotide becomes the point of a rearrangement to *p *:= *N*_young _divided by the sum of all inter-CDS lengths (Model B: *p *:= *N*_young _divided by the sum of all corrected lengths).

We start with the complete set of all *N*_total _inter-CDS regions (assumed to describe the length distribution in the ancestor that divides old from young regions). For each pair, we calculate the probability that it will be broken up in the given time interval as *P *= *p *× length (Model B: effective length); then we draw a random number *r *between 0 and 1 and put the pair into a list of 'surviving' pairs if *r *<*P*. We repeat this simulation until the final list contains *N*_old _regions. The complete simulation is repeated 10 times, and the simulated length distributions shown are averaged over the 10 simulation runs.

## Authors' contributions

WHC assembled the dataset, carried out the analyses and drafted the manuscript. MJL revised the manuscript. WHC, WW and MJL conceived of and designed the study together through iterative discussions, and read and approved the final manuscript.

## Supplementary Material

Additional file 1 **Size distribution of old and young inter-CDS regions for the remaining eight species; this figure is complementary to Figure **1. Click here for file

Additional file 2 **Statistics of the underrepresentation of divergent and uni-directional old pairs in the 11 yeast species**. The NULL expectation here is that if all pairs are distributed randomly, at given inter-CDS range, we would expect 25% of the total pairs to be divergent, 25% to be convergent, and 50% to be co-oriented (uni-directional). Click here for file

Additional file 3 **Reconstruction of phylogenetic relationships of the 11 analysed yeast species, using *Schizosaccharomyces pombe *as an outgroup**. The red star indicates the location of the recent whole genome duplication (WGD). Full species names for the abbreviations are: kla - *Kluyveromyces lactis*, ago -*Ashbya gossypii*, skl - *Saccharomyces kluyveri*, kwa - *Kluyveromyces waltii*, kth - *Kluyveromyces thermotolerans*, zro - *Zygosaccharomyces rouxii*, kpo - *Kluyveromyces polysporus*, cgl - *Candida glabrata*, sca - *Saccharomyces castellii*, sce - *Saccharomyces cerevisiae*, sba - *Saccharomyces bayanus*, spo - *Schizosaccharomyces pombe*. Click here for file

Additional file 4 **Fitting one and two Gaussians to the distributions of inter-CDS distances (log10-transformed) of old, divergently transcribed regions in the 11 analyzed yeast species**. Click here for file

Additional file 5 **Modelling-B of size distribution of inter-CDS regions for the remaining eight species; this figure is complementary to Figure **3. Click here for file

Additional file 6 **Same as Figure **3**& Additional file **5**combined, but showing only results from simulations with 'uncorrected' distances**. Click here for file
