# Supplementary figures and images for: Minimal regulatory spaces in yeast genomes
Source: BMC Genomics. 2011 Jun 16;12:320. doi: 10.1186/1471-2164-12-320 (PMC3128071; doi:10.1186/1471-2164-12-320)

ago

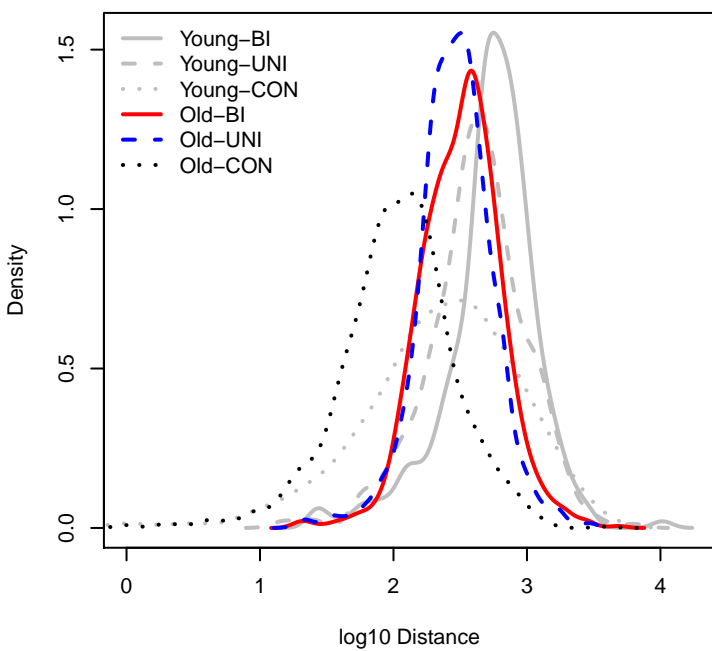

cgl

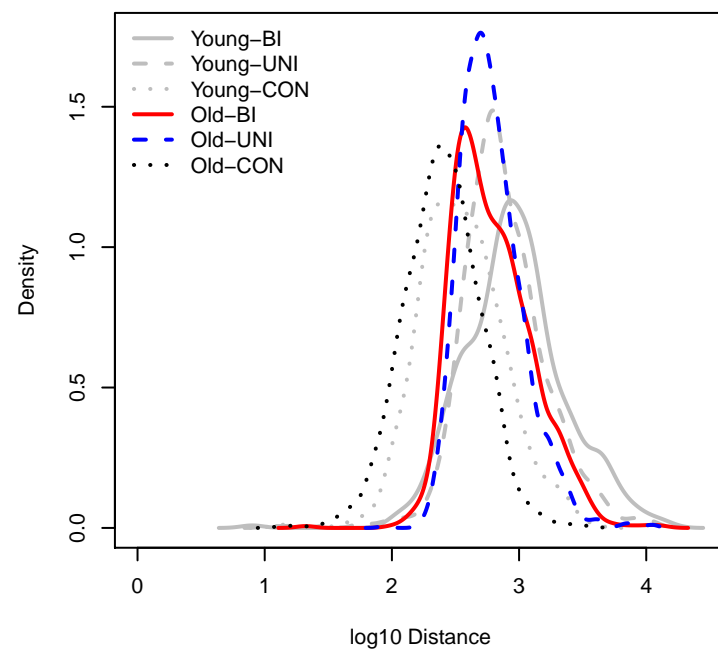

kla

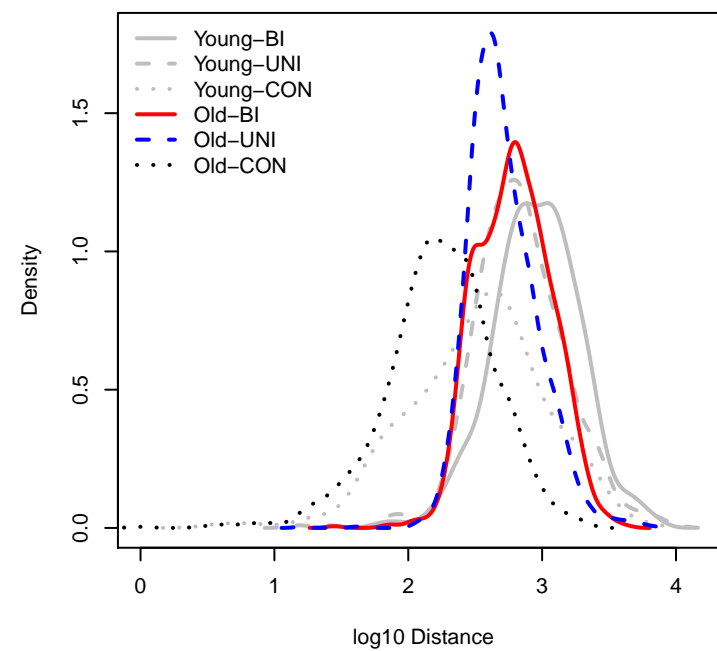

kpo

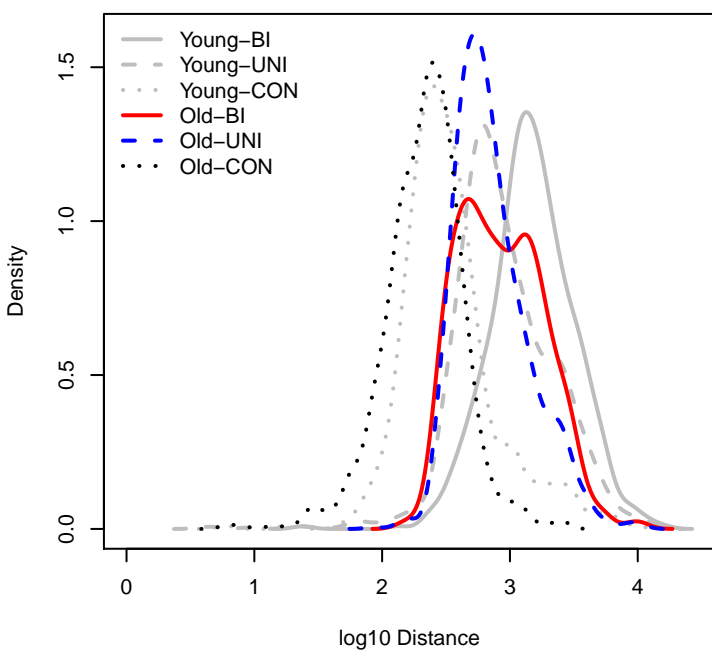

kth

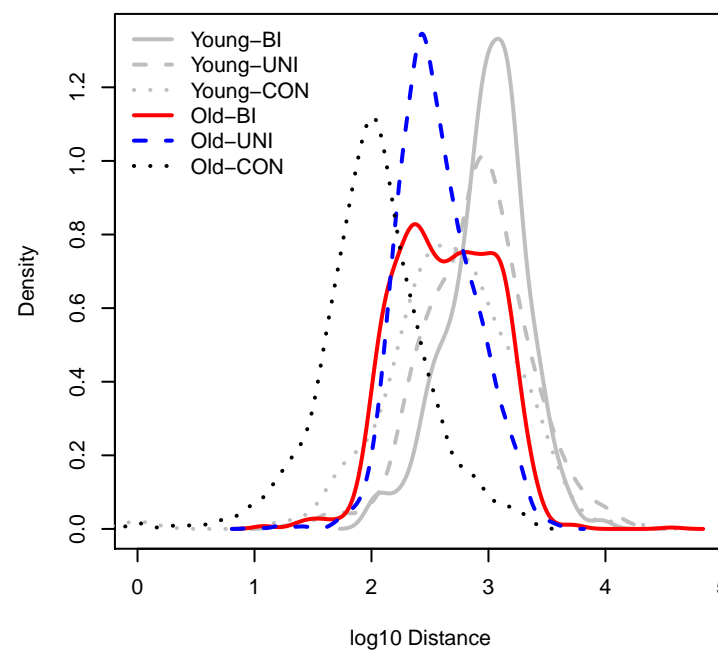

sba

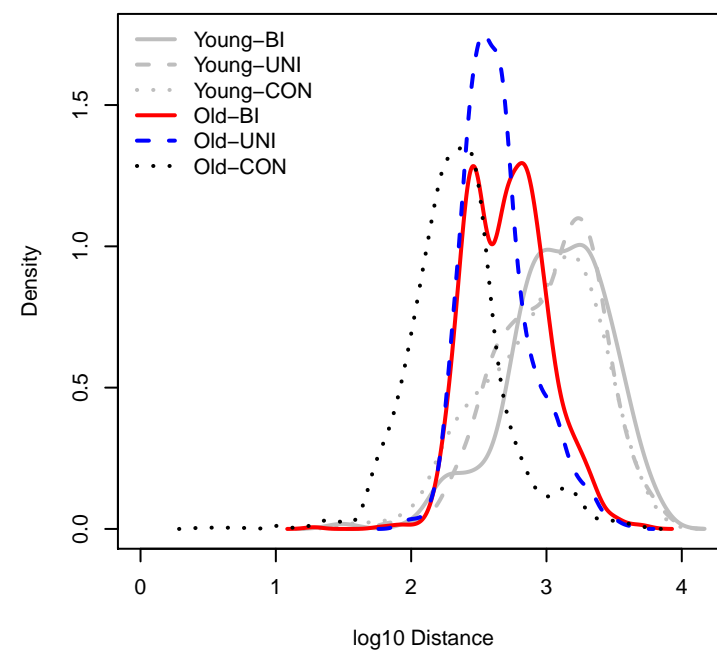

sca

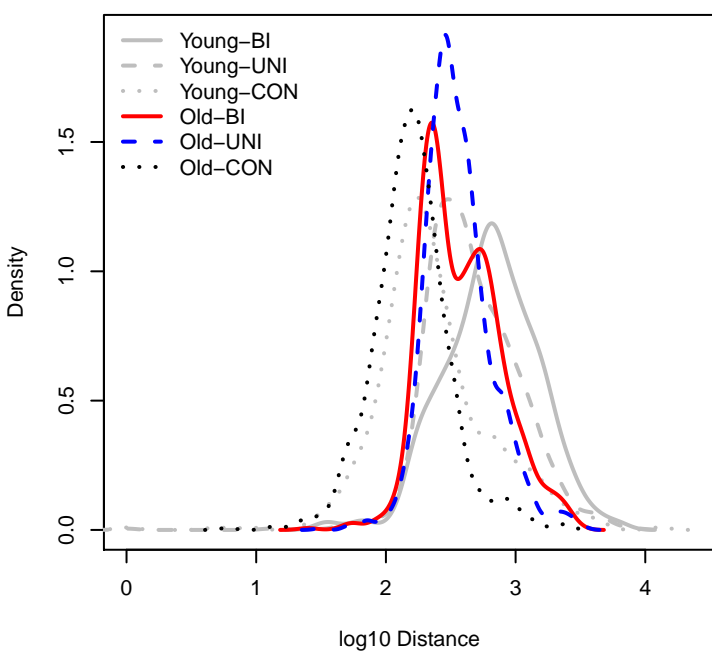

skl

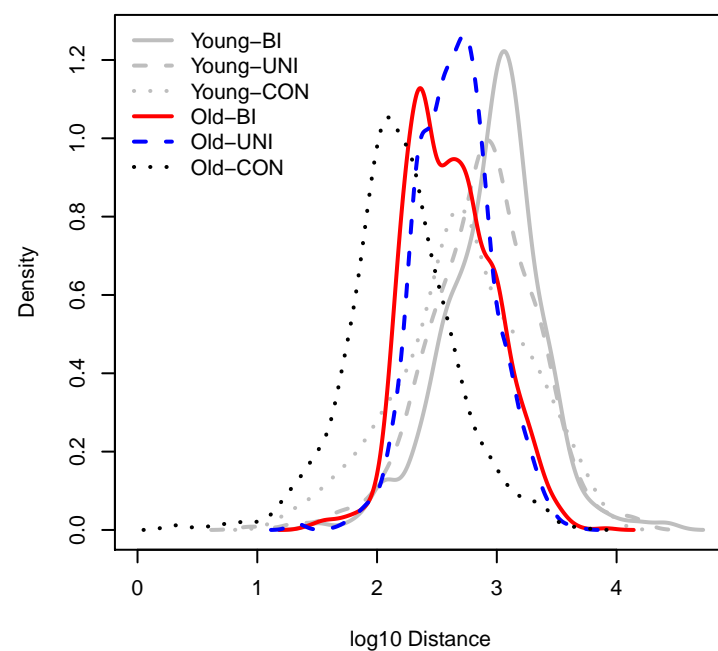

Supplement: Additional file 1 — Size distribution of old and young inter-CDS regions for the remaining eight species; this figure is complementary to Figure 1. [file 1471-2164-12-320-S1.PDF]

0.1

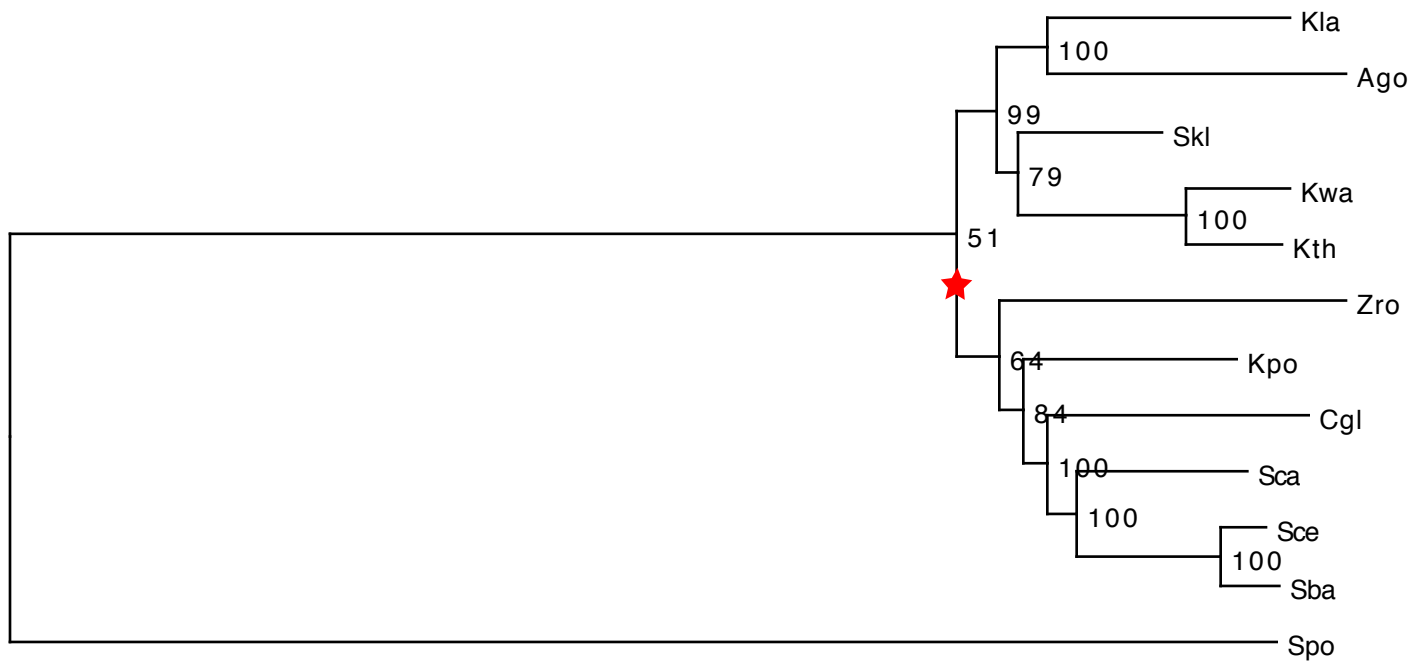

Supplement: Additional file 3 — Reconstruction of phylogenetic relationships of the 11 analysed yeast species, using Schizosaccharomyces pombe as an outgroup. The red star indicates the location of the recent whole genome duplication (WGD). Full species names for the abbreviations are: kla - Kluyveromyces lactis, ago -Ashbya gossypii, skl - Saccharomyces kluyveri, kwa - Kluyveromyces waltii, kth - Kluyveromyces thermotolerans, zro - Zygosaccharomyces rouxii, kpo - Kluyveromyces polysporus, cgl - Candida glabrata, sca - Saccharomyces castellii, sce - Saccharomyces cerevisiae, sba - Saccharomyces bayanus, spo - Schizosaccharomyces pombe. [file 1471-2164-12-320-S3.PDF]

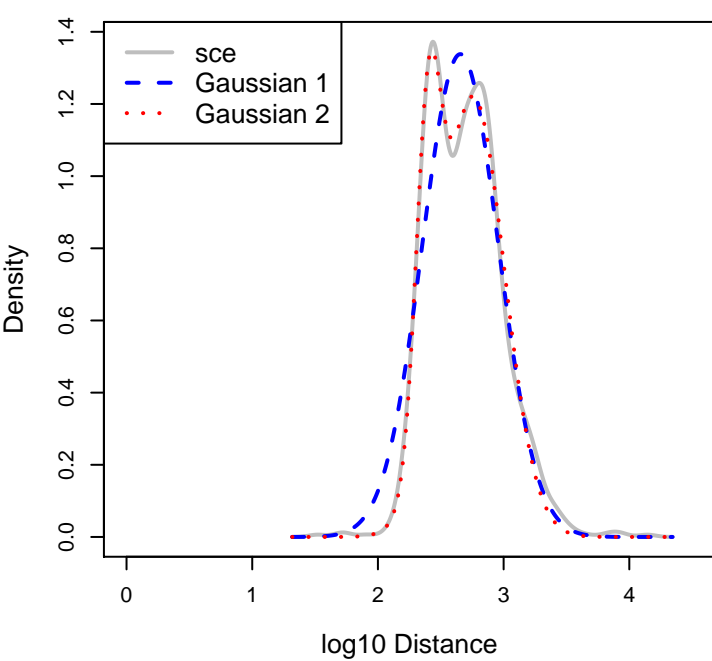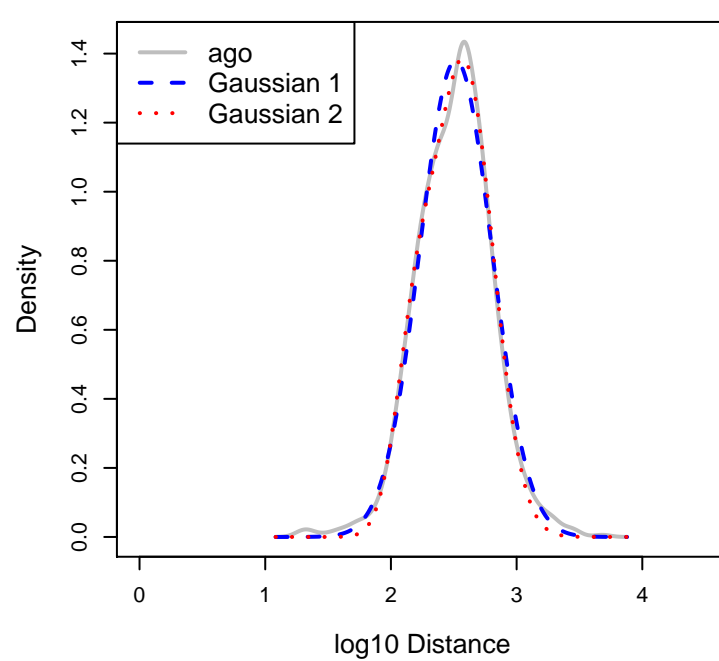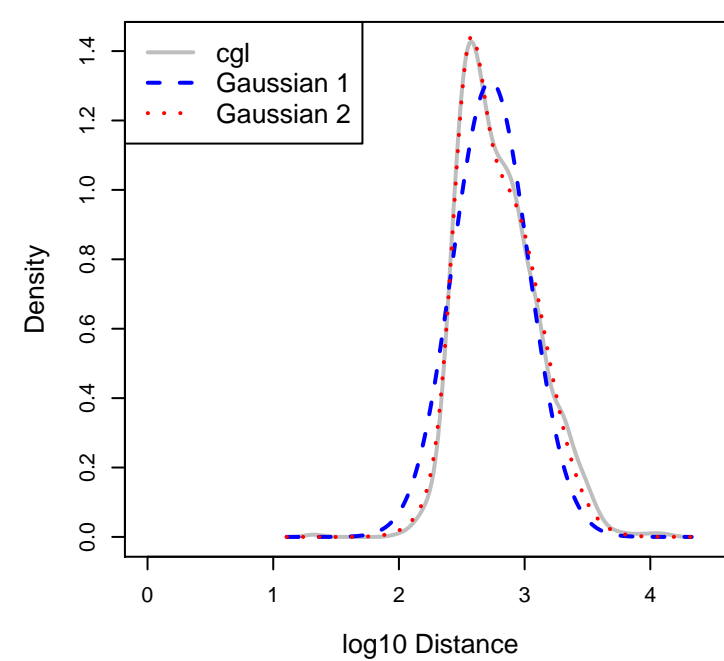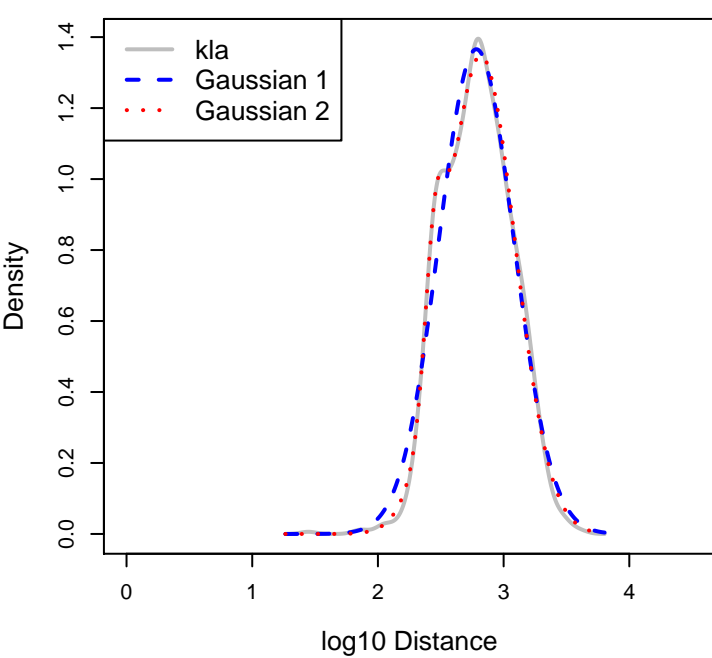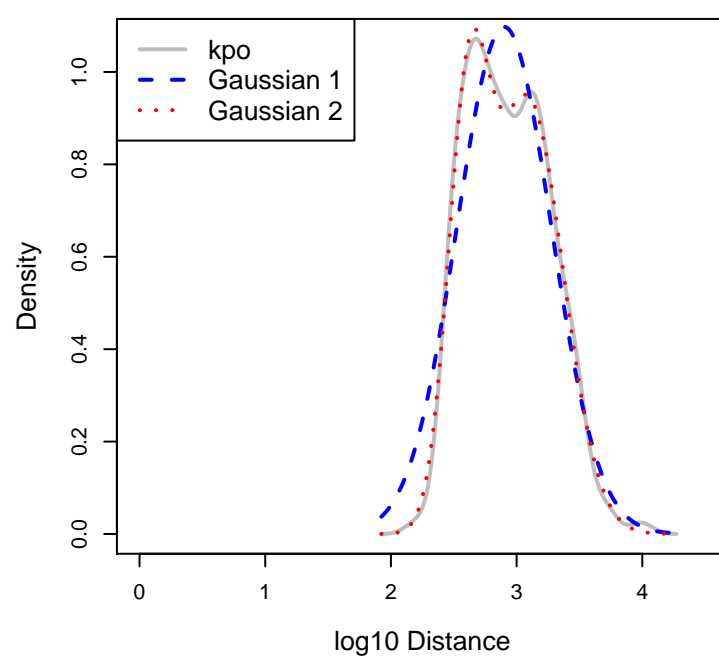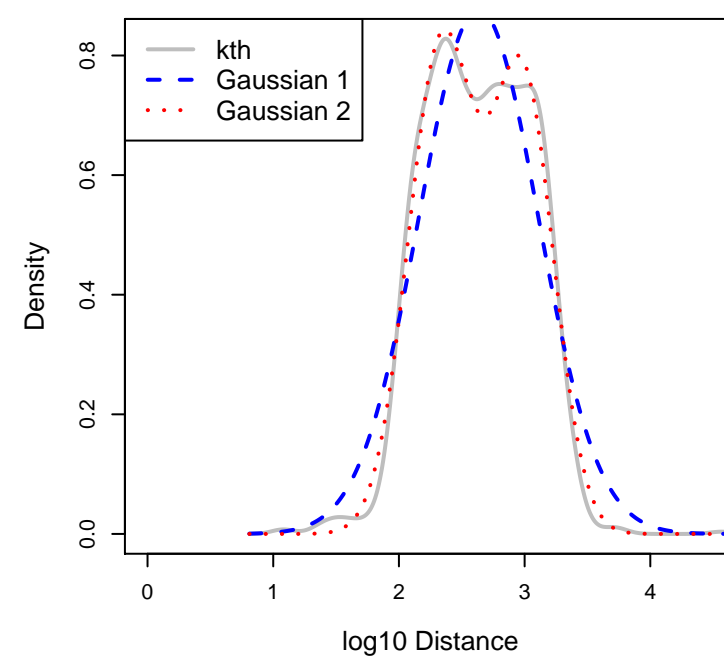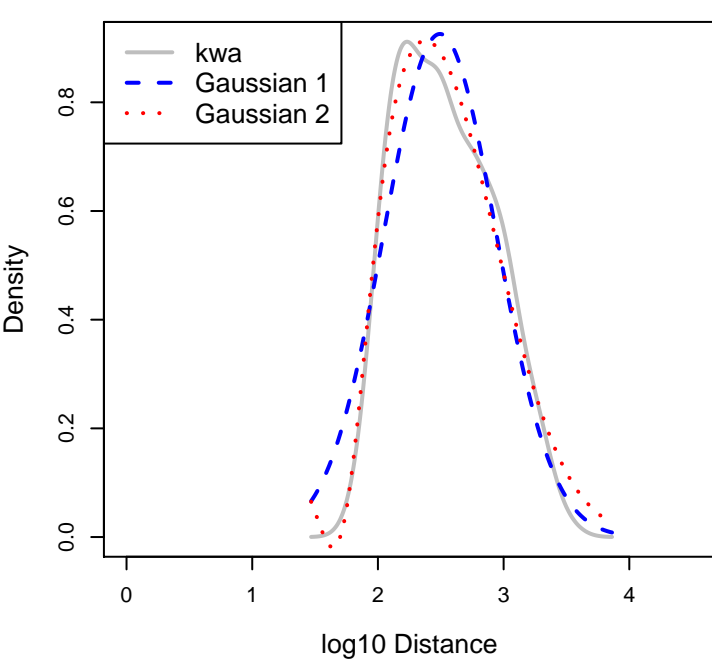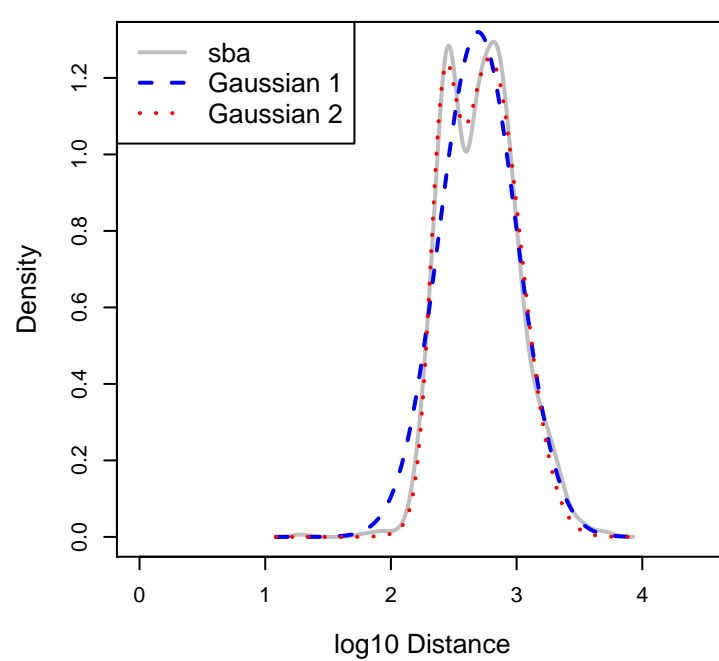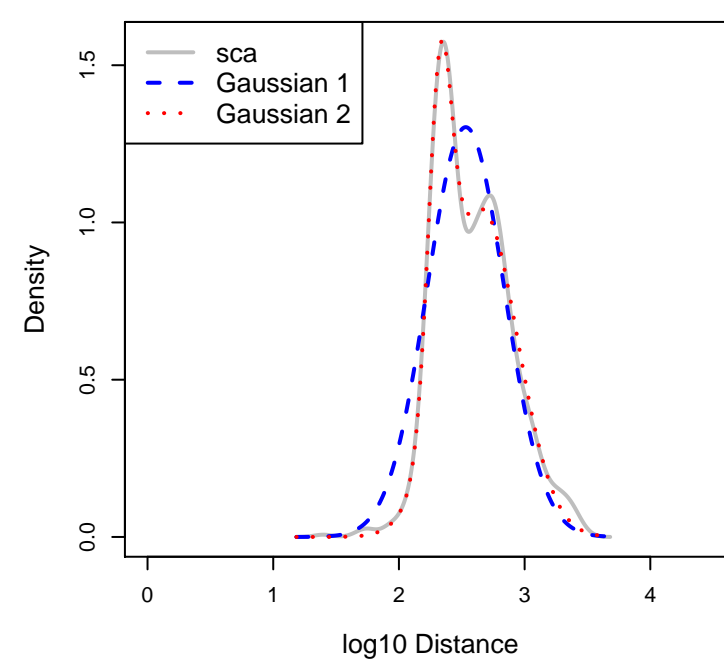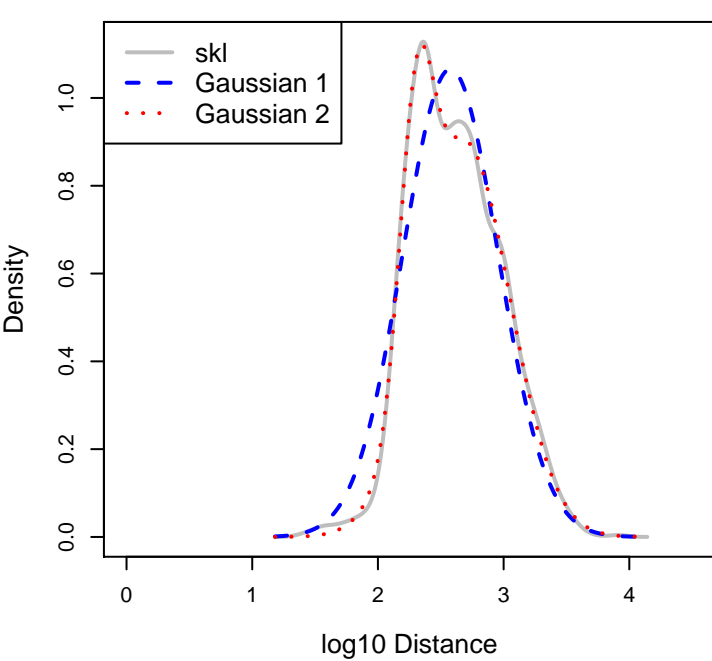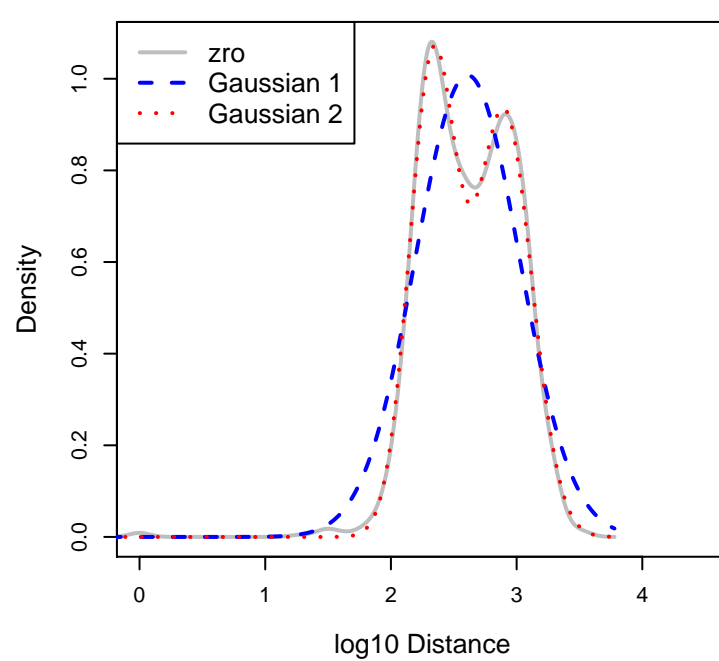

Supplement: Additional file 4 — Fitting one and two Gaussians to the distributions of inter-CDS distances (log10-transformed) of old, divergently transcribed regions in the 11 analyzed yeast species. [file 1471-2164-12-320-S4.PDF]

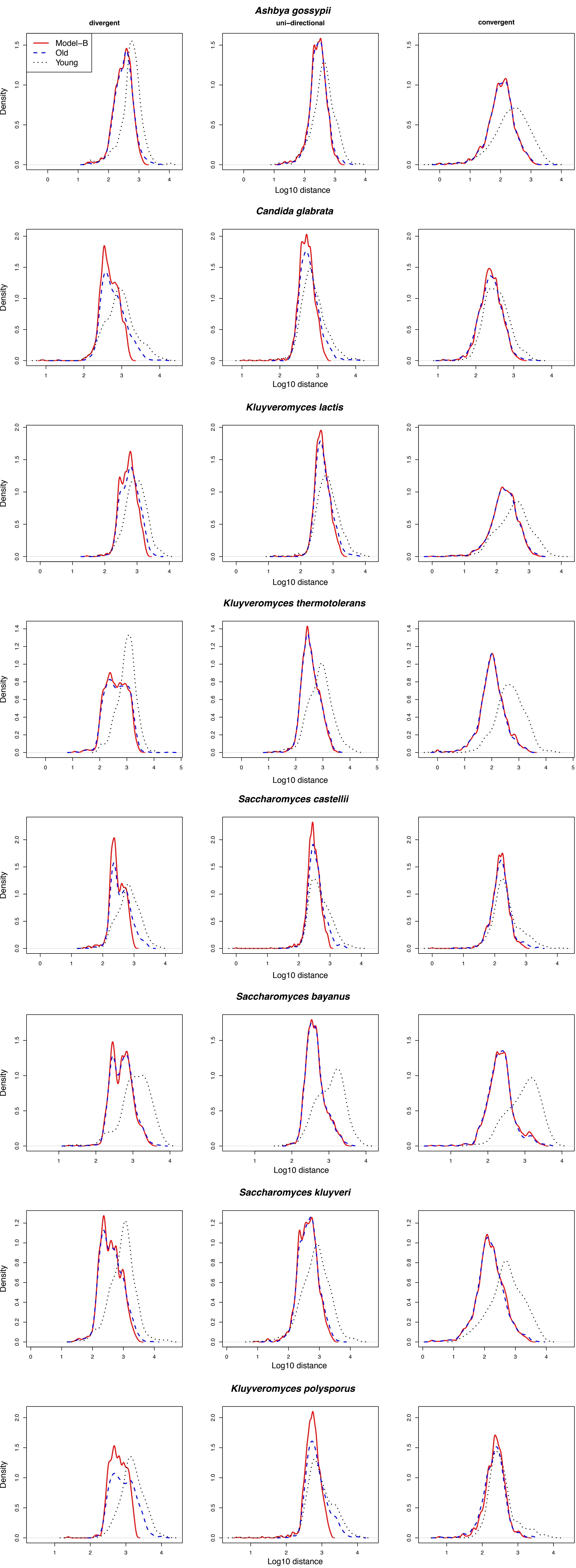

Supplement: Additional file 5 — Modelling-B of size distribution of inter-CDS regions for the remaining eight species; this figure is complementary to Figure 3. [file 1471-2164-12-320-S5.PDF]

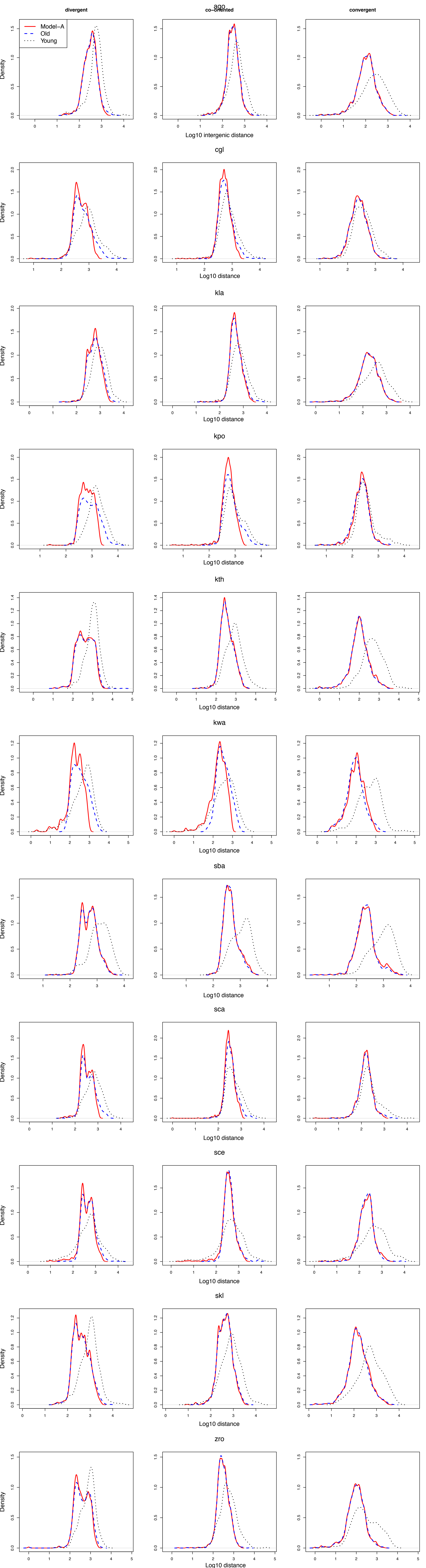

Supplement: Additional file 6 — Same as Figure 3& Additional file 5combined, but showing only results from simulations with 'uncorrected' distances. [file 1471-2164-12-320-S6.PDF]
